# Oxygen Reduction Reaction at Penta-Coordinated Co Phthalocyanines

**DOI:** 10.3389/fchem.2020.00022

**Published:** 2020-01-29

**Authors:** Marco Viera, Jorge Riquelme, Carolina Aliaga, José F. Marco, Walter Orellana, José H. Zagal, Federico Tasca

**Affiliations:** ^1^Departamento de Química de los Materiales, Facultad de Química y Biología, Universidad de Santiago de Chile, Santiago, Chile; ^2^Instituto de Química Física “Rocasolano”, CSIC, Madrid, Spain; ^3^Departamento de Ciencias Físicas, Universidad Andrés Bello, Santiago, Chile

**Keywords:** Co phthalocyanine, penta-coordinated Co phthalocyanines, oxygen reduction reaction, electrocatalysis, volcano correlations

## Abstract

From the early 60s, Co complexes, especially Co phthalocyanines (CoPc) have been extensively studied as electrocatalysts for the oxygen reduction reaction (ORR). Generally, they promote the 2-electron reduction of O_2_ to give peroxide whereas the 4-electron reduction is preferred for fuel cell applications. Still, Co complexes are of interest because depending on the chemical environment of the Co metal centers either promote the 2-electron transfer process or the 4-electron transfer. In this study, we synthetized 3 different Co catalysts where Co is coordinated to 5 N atoms using CoN4 phthalocyanines with a pyridine axial linker anchored to carbon nanotubes. We tested complexes with electro-withdrawing or electro-donating residues on the N4 phthalocyanine ligand. The catalysts were characterized by EPR and XPS spectroscopy. Ab initio calculations, Koutecky–Levich extrapolation and Tafel plots confirm that the pyridine back ligand increases the Co-O_2_ binding energy, and therefore promotes the 4-electron reduction of O_2_. But the presence of electron withdrawing residues, in the plane of the tetra N atoms coordinating the Co, does not further increase the activity of the compounds because of pull-push electronic effects.

**Graphical Abstract:**
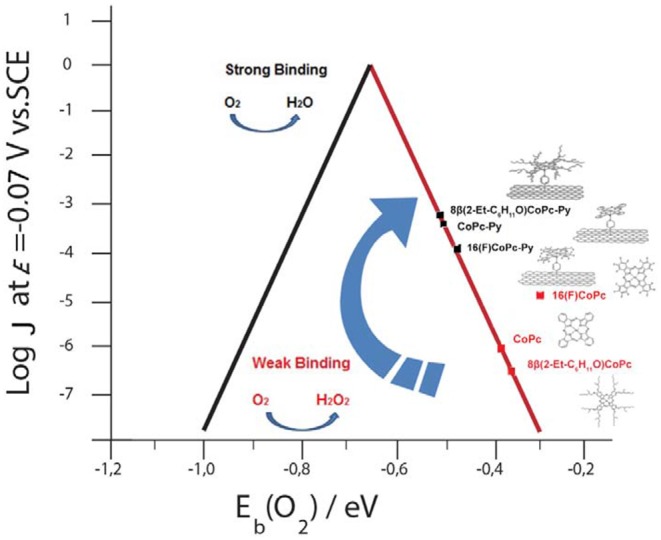
Volcano correlation for penta—coordinated Co phthalocyanines.

## Introduction

Metal phthalocyanines and porphyrins (MPc) belong to the category of macrocyclic complexes where a metallic redox center is coordinated by four pyrrolic nitrogen atoms (MN4). Because of the interesting physical and chemical properties and of the similarities of those molecules to other catalysts found in nature, MN4 have been broadly studied since the early 1960s. In particular, Co phthalocyanine (CoPc) was reported to be an electrocatalysts for the oxygen reduction reaction (ORR) by Jasinski (1964). Later the activity of those complexes was studied for other important reaction like the CO_2_ reduction (Morlanes et al., 2016; Zhang et al., 2017), and the oxidation of L-cysteine (Gulppi et al., 2014), hydrazine (Geraldo et al., 2002, 2008; Venegasa et al., 2017), thiocyanate (Linares-Flores et al., 2012) and thiols (Bedioui et al., 2007) and also for their bio-mimicking activity (Zagal et al., 2010; Venegas et al., 2017; Herrera et al., 2018; Riquelme et al., 2018a).

The search for electrocatalysts for the ORR as active and durable as the rare and costly Pt (which is the industrial standard for this reaction) is still pushing the search for a non-precious metal catalyst. The difficulties at finding an appropriate catalyst arise from the complexity of the reaction the lower activity and stability. From the moment that up to 4 electrons and 4 protons are necessary for the complete reduction of dioxygen various intermediates can be formed during the process accounting for the so-called “scaling relationship” (Nørskov et al., 2004; Koper, 2011; Christensen et al., 2016; Katsounaros and Koper, 2017).

For example, in alkaline media, the reaction might proceed via a 2-electron transfer process (following reaction 1) with the release of ${\text{HO}}_{2}^{-}$ or via a 4-electron transfer (following reaction 2) leading to formation of H_2_O with the splitting of the O-O bond.

2-electron transfer ORR in basic media:

(1) $${\text{O}}_{2}+{\text{H}}_{2}\text{O}+2{\text{e}}^{-}\to {\text{HO}}_{2}^{-}+{\text{OH}}^{-}\;{\text{E}}_{0}=-0.076\text{ V}\;\text{vs}.\;\text{NHE}$$

4-electron transfer ORR in basic media:

(2) $$\begin{array}{c} {\text{O}}_{2}+2{\text{H}}_{2}\text{O}+4e^{-}\to 4{\text{OH}}^{-}\;\;{\text{E}}_{0}=0.401\text{ V}\;\text{vs}.\;\text{ NHE} \end{array}$$

For the 4-electron transfer process the intermediates are:

(3) $${\text{O}}_{2}+{\text{e}}^{-}⇌{\text{O}}_{2}^{-}{\;}_{\text{ad}}$$

(4) $${\text{O}}_{2}^{-}{\;}_{\text{ad}}+{\text{H}}_{2}\text{O}+{\text{e}}^{-}⇌{\text{HO}}_{\text{2​ }}^{-}{}_{\text{ad}}\text{+ }{\text{OH}}^{-}$$

(5) $${\text{HO}}_{2}^{-}{\;}_{\text{ad}}+{\text{H}}_{2}\text{O}⇌{\text{2OH}}_{\text{ad}}+{\text{OH}}^{-}$$

(6) $$\begin{array}{r} \text{O}{\text{H}}_{\text{ad}}+{\text{e}}^{-}⇌\text{ O}{\text{H}}^{-} \end{array}$$

Depending on the pathway of the reaction we might have 2 or 3 intermediates bound to the active sites as ${\text{O}}_{2}^{-}{\;}_{\text{ad}}$, ${\text{HO}}_{2}^{-}$ and OH_ad_ or dissolved ${\text{HO}}_{2}^{-}$ if the reaction proceeds as in Equation (1).

The ORR catalyzed at electrodes modified with CoN4 complexes is of major interest because of the ease and the convenience of the synthesis of those redox complexes and because of the different catalytic properties of the various complexes which were synthetized to optimize the catalysis. For example ORR catalyzed by unsubstituted CoPc or by CoPc with the addition of one or more electron-donating or electron-withdrawing substituents adsorbed directly on graphite proceeds via a 2-electron transfer process (Zagal et al., 1992; Sun et al., 2014; Zagal and Koper, 2016; Ye et al., 2017; Abarca et al., 2019) whereas complexes with pendant residues that increase the electron back-bonding would promote a direct 4-e process as in Equation (2) (Steiger and Anson, 1995; Tse et al., 1997; Riquelme et al., 2018a,b). Co-cofacial bimetallic complexes and dimeric Co porphyrins that bind simultaneously both dioxygen atoms would also transfer directly 4 electrons to O_2_ (Collman et al., 1980; Durand et al., 1983; Le Mest et al., 1997; Peljo et al., 2012; Swavey and Eder, 2013; Wada et al., 2013; Dey et al., 2017; Oldacre et al., 2018). Penta-coordinated complexes involve a Co center coordinated to 4 planar N atoms and an axial N belonging to a pyridine molecule as for example in VB12 seem to promote a 4-e process. CoPc-Py-CNT complex also promotes the reduction of dioxygen following a mixed 2 and 4-electrons process (Zagal et al., 2010; Fukuzumi et al., 2018; Riquelme et al., 2018a; Zhou et al., 2019). Recently, Zhou and collaborators studied the effect of various axial ligands on the activity of Co porphyrin toward the ORR (Zhou et al., 2019). They found that an increased coordination strength of the axial ligand leads to higher activity of the catalyst (Zhou et al., 2019). Because of the presence of the fifth coordination the d orbitals of the Co redox center would become more similar to the orbitals of O_2_ and therefore Co would form a stronger bond with O_2_. It is important to point out that in correlations of activity vs. the M-O_2_ binding energy, volcano correlations are obtained using a great number of MN4 complexes of Cr, Mn, Fe, Co, and Cu as central metals. Strongly O_2_ binding complexes lye on one side of the volcano and weakly binding complexes lye on the other side of the volcano. Most CoN4 complexes, including CoPc appear on the weak binding side. This indicates that the activity of these CoN4 catalysts increases as the Co-O_2_ binding energy increases. It was shown for planar CoN4 complexes, that stronger electron withdrawing planar residues would reduce the distance between the O_2_ and Co orbitals increasing as the Co-O_2_ binding energy (Van Den Brink et al., 1983; Zagal et al., 1992).

What happens in the presence of fifth axial coordination? How does the fifth coordinated N affect the Co-O_2_ binding energy of CoN4? How does it affect the activity of CoN4 complexes toward the ORR when in the presence of ligands in the plane of the phthalocyanine ring? Can a linear correlation be reproduced as for the planar catalysts?

To answer those questions unsubstituted CoPc, perfluorinated CoPc (16(F)CoPc), and Cobalt-octaethylhexyloxyphthalocyanine (8(2-Et-C_6_H_11_O)CoPc) were coordinated to CNT modified with covalently attached pyridine moieties. Their activities at modified rotating ring disk electrodes were compared to that of the planar catalysts physically adsorbed on unmodified CNT. EPR and XPS spectroscopy analysis confirm the changes of the electronic structure of the Co redox center while DFT calculations provide the O_2_-Co binding energy of the complexes. Stronger Co-O_2_ binding energy leads to higher ORR catalytic activity.

## Experimental

### Modification of Co Phthalocyanines

Cobalt phthalocyanine (CoPc), cobalt-hexadecafluorophthalocyanine (16(F)CoPc), and 4-aminopyridine (Py), 2,2-diphenyl-1-picryl- hydrazyl (DPPH), N,N-dimethylformamide (DMF), isopropyl alcohol, NaOH, HCl, K_2_HPO_4_ were obtained from Sigma (St. Louis, USA). Cobalt-octaethylhexyloxyphthalocyanine (8(2-Et-C_6_H_11_O)CoPc), was synthesized according to the literature (Weber and Busch, 1965). CNT were from Nanocyl (Sambreville, Belgium). Previously to modification the CNT were The functionalization of the CNT with Py was obtained using the diazonium reaction where 0.1 g of CNT dispersion was added to 5 g of NaNO_2_, 7 g of Py, and 1 g of DPPH 5 ml of 4 M HCl water solution; DPPH was used as radical scavenger to prevent the formation of multi-layers (Menanteau et al., 2016). The Py-CNT were washed with isopropanol and water, filtered and dried in a furnace at 80°C. Next, the Py-CNT were suspended in DMF and modified with either CoPc, or with 16(F)CoPc, or with 8(2-Et-C_6_H_11_O)CoPc by refluxing in N_2_ at 150**°**C to obtain CoPc-Py-CNT, 16(F)CoPc)-Py-CNT, and 8(2-Et-C6H11O)CoPc-Py-CNT. To modify electrodes an ink of the modified phthalocyanine was obtained by dispersing 1 mg of the complex in 1 ml of a mixture of 25% volume isopropyl alcohol and 75% H_2_O. Finally, 20 μl of ink (final load 0.1 mg cm^−2^) was dropped onto the surface of the electrode and let to dry.

### Electrochemical Characterization

Origalys (Rillieux-la-Pape, France) KCl saturated Ag|AgCl was used as reference electrode and all the potentials are reported using this reference. As working electrode, a rotating ring disk electrode (RRDE) from Pine Instruments (Durham, NC, USA) was used. An edge-plane pyrolytic graphite disk (5 mm diameter and 4 mm thick), was mounted into a Teflon rotating shaft containing the Pt ring electrode (external diameter of 7.50 mm, internal diameter 6.50 mm). The MSR rotator was also from Pine Instruments (Durham, NC, USA). 800 grit emery paper was used to renew the graphite disk. An Autolab PGSTAT 302N with a dual mode bipotentiostat module (Utrecht, The Netherlands) was used to perform electrochemical experiments. During linear sweep voltammetry experiments the ring potential was set to 0.65 V vs. Ag|AgCl.

### EPR and XPS Spectroscopy

Dried powder CNT and of each pure and modified complex was used for EPR and XPS spectra. EPR spectra were collected at 298 K with a Bruker EMX-1572 spectrometer working at 9.39 GHz (X-band), using powder samples. XPS spectra were recorded at the Recasolano center using a vacuum of ca. 3 × 10^−9^ mbar, a PHOIBOS-150 electron analyzer (SPECS), Al Kα radiation (1486.6 eV, 100 W) and constant pass energy of 20 eV. The binding energy scale was referenced at the main C 1s signal, which was set at 284.6 eV.

### Theoretical *ab-initio* Calculations

The Quantum-ESPRESSO *ab initio* package was used to perform spin-polarized density functional theory (DFT) calculations (Giannozzi et al., 2009). Dispersive interactions were included by the van der Waals functional (vdW-DF2) into the exchange-correlation potential (Berland et al., 2015). Kohn-Sham eigen-functions were expanded on a plane-waves basis set where the interaction between valence electrons and ion cores were described by ultrasoft pseudo-potentials (Garrity et al., 2014). Converged results have been achieved by using cut-off energies of 35 Ry on plane waves and of 280 Ry on the electronic density. We consider an (8,8) single-walled carbon nanotube (CNT) with 11 Å in diameter. Calculations were conducted in large unit cells with periodic boundary conditions along the CNT axis, including a vacuum region of 15 Å. The sampling in the irreducible part of the Brillouin zone was restricted to the Γ point. The systems were fully relaxed until the force in each atom component was <0.025 eV/Å. The activation energy for the O_2_ dissociation was obtained by calculating the minimum energy path using the Nudged Elastic Band (NEB) method (Henkelman et al., 2000), considering 10 images in the reaction coordinate.

## Results and Discussion

**Figure 1:**
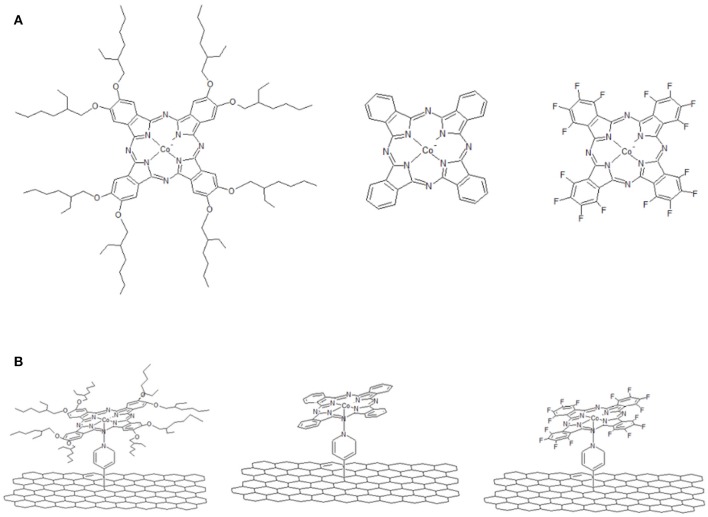
**(A)** Schematic representation of CoN4. From left to the right: 8(2-Et-C_6_H_11_O)CoPc, CoPc, and 16(F)CoPc. **(B)** Schematic representation of CoN5. From left to the right: 8(2-Et-C_6_H_11_O)CoPc-Py-CNT, CoPc-Py-CNT, and 16(F)CoPc-Py-CNT.

In Figure 1 we report a schematic representation of CoPc, 16(F)CoPc and 8(2-Et-C_6_H_11_O)CoPc molecules (Figure 1A) and the corresponding molecule coordinated to the pyridine modified CNT (i.e., CoN4-Py-CNT) (Figure 1B). The modification of CNTs with pyridine moieties to form Py-CNT has been reported previously (Bahr and Tour, 2001; Tasca et al., 2011; Tuci et al., 2014; Venegas et al., 2017; Riquelme et al., 2018a). Also, the modification of CoN4 with pyridine or similar strongly coordinating solvents has been shown before (Cariati et al., 1975; Liao and Scheiner, 2001; Riquelme et al., 2018a). Nevertheless, the synthesis and the studies of the ORR at substituted penta-coordinated CoPc have not been reported before.

### EPR and XPS Characterization

**Figure 2:**
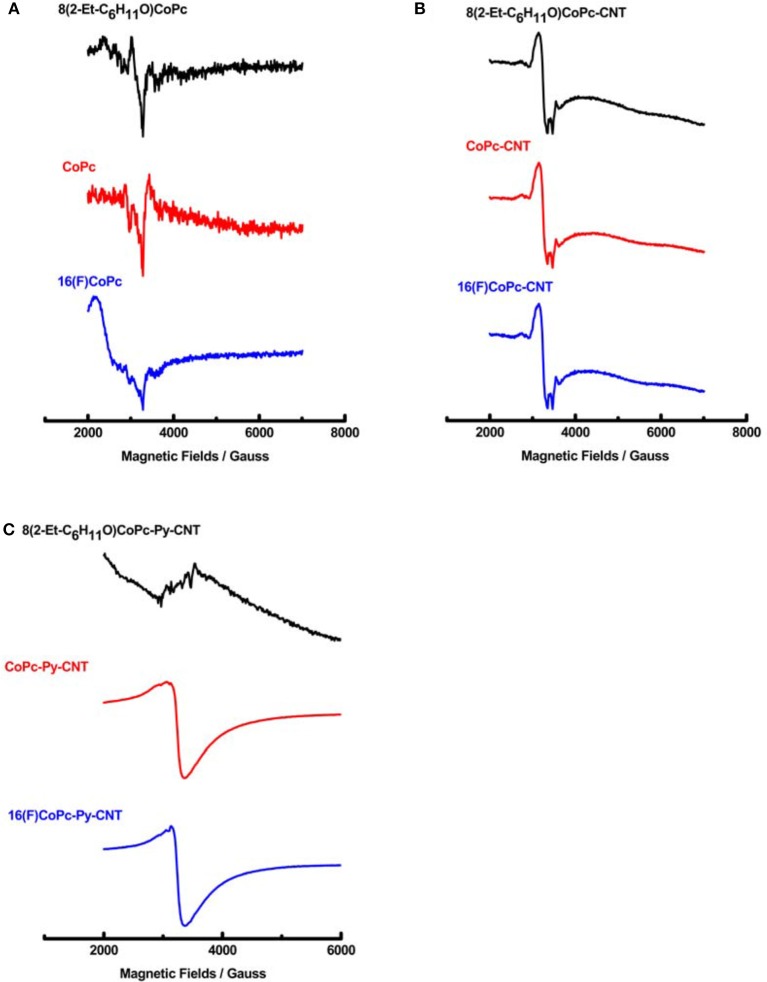
**(A)** EPR spectra of 8(2-Et-C_6_H_11_O)CoPc, CoPc, and 16(F)CoPc. **(B)** EPR spectra of 8(2-Et-C_6_H_11_O)CoPc-CNT, CoPc-CNT, and 16(F)CoPc-Py-CNT. **(C)** EPR spectra of 8(2-Et-C_6_H_11_O)CoPc-Py-CNT, CoPc-Py-CNT, and 16(F)CoPc-Py-CNT.

To obtain insights into the redox state and the spin state of the d electron in CoN4 and CoN5 complexes, EPR and XPS spectra of the complexes were obtained. The EPR spectra of 8(2-Et-C_6_H_11_O)CoPc, CoPc, and 16(F)CoPC molecules do not present strong EPR signals at room temperature but only features at around 2000 G corresponding to the presence of the low spin form of Co(II) (Figure 2A). Co phthalocyanines EPR spectra were previously studied in details by Assour (1965), and Kontarinis et al. (2003). The EPR spectrum of the complexes in the presence of CNT, are shown in Figure 2B. CNT powder has a strong signal due to defects in the graphene structure (Corzilius et al., 2007; Riquelme et al., 2018a) with a *g* factor value of 2.055 (Riquelme et al., 2018a). The signal of CNT modified with the various Co phthalocyanines, varies in each sample (Figure 2B), indicating the electronic interaction between the CNT and the adsorbed moieties. When in the presence of the pyridine modified CNT, the *g* factor value of the CoN5 complexes drastically decreases because of the new chemical environment with the exception of sample 8(2-Et-C_6_H_11_O)CoPc-Py-CNT where no EPR signal is observed because of a high spin (S = 3/2) Co(II) specie (Figure 2C). The EPR spectrum of the penta-coordinated compounds CoPc-Py-CNT and 16(F)CoPC-Py-CNT presents a value of *g*_iso_ around 2.08 consistent with a Co(III) in a low spin state (LS) S = 1/2, where the electron is occupying a ${\text{d}}_{\text{z}}^{2}$ type orbital or a Co(II) in a low spin configuration S = 1/2. The hyperfine structures were not observed at room temperature (Baumgarten et al., 2001).

The interpretation of the Co 2p XPS data in systems like these is not straightforward. Low spin (S = 0) diamagnetic Co(III) and high spin (S = 3/2) paramagnetic Co(II) compounds show very characteristic spectra (Briggs and Gibson, 1974; Frost et al., 1974; Oku, 1978). In the case of low spin (S = 1/2) Co(II) complexes, the Co 2p spectra show very subtle differences with respect to the spectra pertaining to low spin (S = 0) Co(III) and care must be taken when carrying out the corresponding analysis. The less usual intermediate spin (S = 1) Co(III) complexes have also very distinctive features (Oku, 1978). The binding energies of the main photoemission lines is not always indicative of the oxidation state or the changes in charge distribution of cobalt since they can be affected by the type of ligands or the spin state (Briggs and Gibson, 1974). Therefore, it is better to resort to other spectral features as the presence or absence of shake-up satellite structure (which inform on the paramagnetic or diamagnetic nature, respectively, of the cobalt ion), the occurrence of multiplet splitting and the energy separation, ΔE, between the Co 2p3/2 and Co 2p1/2 core level lines. Thus, low spin (S = 0) diamagnetic Co (III) complexes show Co 2p spectra with narrow, quite symmetric lines, the absence of multiplet splitting peaks because Co(III) (S = 0) has no unpaired electrons, almost total absence of satellites except for a weak one (interpreted as an energy loss feature) at about 10 eV above the main Co 2p3/2 line and a ΔE = 15.0–15.1 eV (Briggs and Gibson, 1974; Frost et al., 1974; Oku, 1978). On the contrary, paramagnetic (S = 3/2) high spin Co(II) ions present Co 2p spectra with broad and asymmetric lines due to the occurrence of multiplet splitting, intense shake-up satellite structure ca. 5.6–6.0 eV above the main Co 2p3/2 line and a ΔE = 16.0 eV (Briggs and Gibson, 1974; Frost et al., 1974). The Co 2p spectra of intermediate spin (S = 1) Co (III) complexes may have asymmetric lines because of multiplet splitting, satellites at about 3.2–4.2 eV above the main Co 2p3/2 photoemission line and a ΔE intermediate between 15.0 and 16.0 eV (Oku, 1978). Finally, low spin (S = 1/2) Co(II) complexes give Co 2p spectra with very weak satellites but having multiplet splitting peaks about 2.0 eV above the main Co 2p3/2 line which are displayed as a shoulder or asymmetry of that peak. For these compounds, the value of ΔE is also intermediate between 15.0 and 16.0 eV (Briggs and Gibson, 1974; Frost et al., 1974). An interesting observation is that in low spin (S = 1/2) Co(II) multiplet splitting peaks are observed mainly close to the Co 2p3/2 peak, but are less evident, or simply inexistent, close to the Co 2p1/2 peak (Briggs and Gibson, 1974). However, shake up satellites appear on the high binding energy side of both photoemission lines.

**Figure 3:**
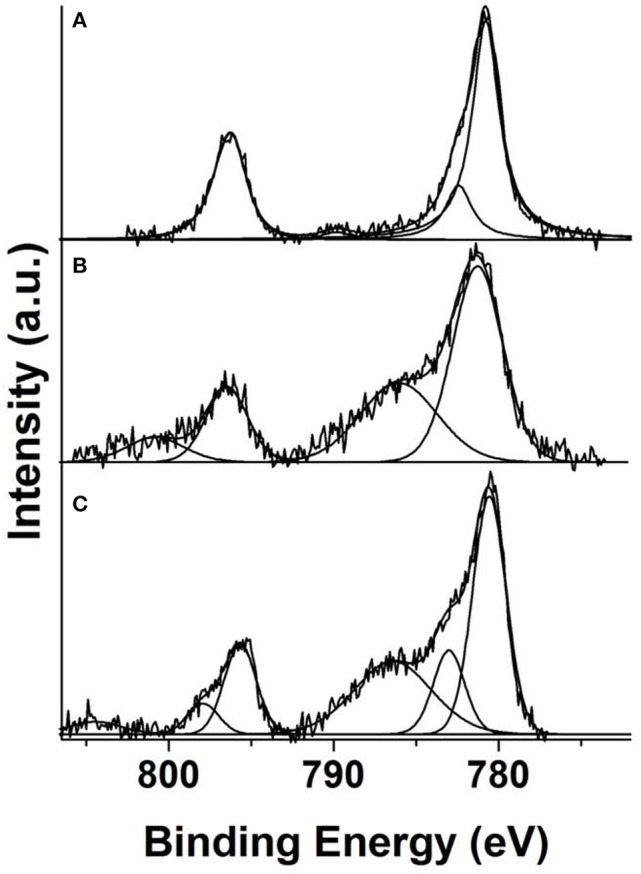
Deconvoluted Co 2p XPS spectra recorded from powder samples of **(A)** 8(2-Et-C_6_H_11_O)CoPc-Py-CNT, **(B)** CoPc-Py-CNT, **(C)** 16(F)CoPc-Py-CNT.

Having all this information in mind we can face now the interpretation of the Co 2p spectra recorded from 8(2-Et-C_6_H_11_O)CoPc-Py-CNT, CoPc-Py-CNT, and 16(F)CoPc-Py-CNT and which are shown in Figures 3A–C, respectively. The spectrum recorded from CoPc-Py-CNT shows a relatively narrow doublet with a binding energy of the Co 2p3/2 core level of 780.8 eV and ΔE = 15.5 eV. The Co 2p3/2 line is clearly asymmetric in its high binding energy side having a clear shoulder at 782.3 eV, i.e., 1.7 eV above the binding energy of the mentioned line. This shoulder, as stated above, is compatible with the occurrence of multiplet splitting and, therefore, compatible with a low spin (S = 1/2) Co(II) species. The Co 2p spectrum recorded from 16(F)CoPc-Py-CNT is very different. It shows much broader lines and the presence of very intense shoulders above the main photoemission lines. Since these shoulders appear close to both core level lines they must correspond with shake-up satellites. The Co 2p3/2 peak is located at 781.2 eV (ΔE = 15.3 eV) and its corresponding satellite at 785.4 eV, i.e., a separation of 4.2 eV. All these values support the assignment of this spectrum to an intermediate spin (S = 1) Co(III) species. Finally, the Co 2p spectrum recorded from 8(2-Et-C_6_H_11_O)CoPc-Py-CNT is also different from the spectra recorded from the other two samples. In this spectrum the photoemission lines are clearly asymmetric having shoulders in their high binding energy side (~2.4 eV) what is indicative of the occurrence of multiplet splitting. The spectrum also shows the presence of a clear and intense shake-up satellite at 786.5 eV, i.e., 6.0 eV above the main Co 2p3/2 peak which is located at 780.5 eV. All this allows, with no doubt, to identify the Co ion in this compound as a high spin (S = 3/2) Co(II) species.

### DFT Calculations

**Figure 4:**
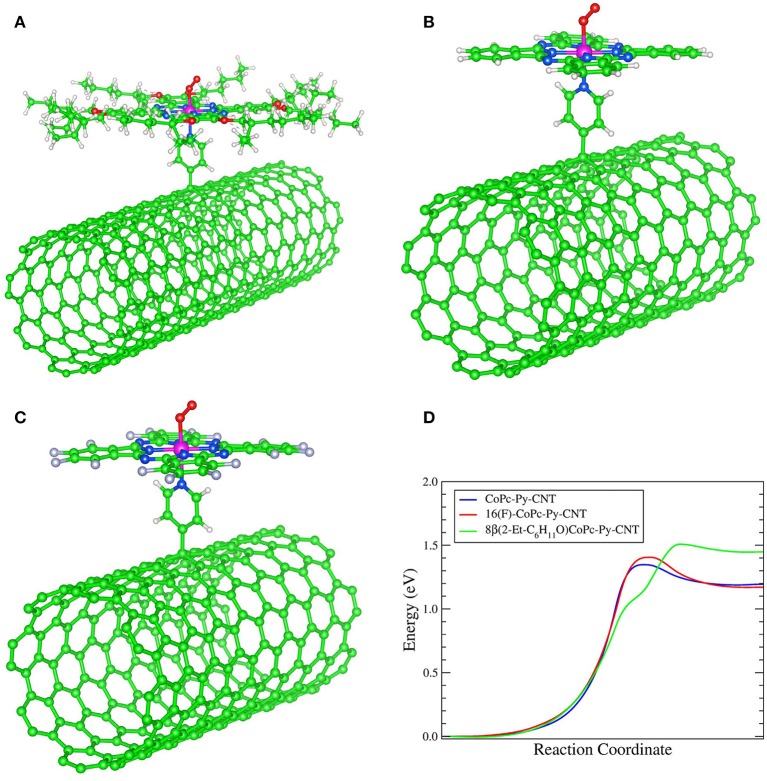
Equilibrium geometry of O_2_ adsorbed in the end-on configuration on the Co center of: **(A)** 8(2-Et-C_6_H_11_O)CoPc-Py-CNT, **(B)** CoPc-Py-CNT and **(C)** 16(F)CoPc-Py-CNT. **(D)** Minimum energy path for the *O_2_ dissociation on the Co center in 8(2-Et-C_6_H_11_O)CoPc-Py-CNT, CoPc-Py-CNT, and 16(F)CoPc-Py-CNT.

We performed spin-polarized DFT calculations to simulate the structure of the CoN5, the binding energy of O_2_ to Co redox centers, and the activation energy for the ORR. In Figures 4A–C we show the stable geometries of the O_2_ molecule adsorbed on the Co metal center of 8(2-Et-C_6_H_11_O)CoPc-Py-CNT, CoPc-Py-CNT, and 16(F)CoPc-Py-CNT. For the three complexes, O_2_ is found to bind in the end-on configuration. The total energy was calculated for the allowed spin state in order to find the most stable spin configuration. Our results show that all the complexes in their equilibrium geometry exhibit a low-spin configuration, showing a magnetic moment *m* = *1* μ_*B*_, that is with an unpaired electron (S = 1/2). After approaching at the CoN5 center, the O_2_ molecule attaches to the Co atom with one O atom with binding energies of −0.595, −0.532, and −0.524 eV for CoPc-Py-CNT, 16(F)CoPc-Py-CNT, and 8(2-Et-C_6_H_11_O)CoPc-Py-CNT, respectively. Interestingly, the equilibrium spin state of the complexes with the adsorbed O_2_ molecule shows different spin configurations with *m* = *1* μ_*B*_ for CoPc-Py-CNT and *m* = *2* μ_*B*_ for 16(F)CoPc-Py-CNT, and 8(2-Et-C_6_H_11_O)CoPc-Py-CNT. In Table 1 we report the equilibrium geometry of O_2_ attached to the Co center for the three compounds. We observe that for O_2_-CoPc-Py-CNT, the O-Co bond distances are shorter than those found in O_2_-16(F)CoPc-Py-CNT and O_2_-8(2-Et-C_6_H_11_O)CoPc-Py-CNT, which indicate that the CoPc border modifications tend to weaken the O_2_-Co bond, consistent with the smaller O_2_ binding energy, as reported in Table 2. Moreover, the distance between the Co atom and the N axial ligand [N(a)-Co] in O_2_-CoPc-Py-CNT is shorter than in the other compounds. This suggests a higher electronic localization at the Co center, which may explain the higher magnetic moment observed in O_2_-CoPc-Py-CNT. We also studied the activation energy for the O_2_ dissociation after adsorption on the Co center by calculating the minimum energy path of the reaction using the NEB method. Figure 4D shows the results for the three complexes. The calculated activation energies (the height of the barriers) range between 1.35 to 1.51 eV, where CoPc-Py-CNT would show the lowest value, close to 16(F)CoPc-Py-CNT. If we associate lower activation energy with higher catalytic activity, the ORR catalytic activity for the complexes under study would follow the trend CoPc-Py-CNT ≥ 16(F)CoPc-Py-CNT > 8(2-Et-C_6_H_11_O)CoPc-Py-CNT. Anyway, the activities of the three compounds do not vary much. Table 2 compares our results for O_2_ binding and activation energies for the three complexes. We observe a clear correlation between higher O_2_ binding energies with lower O_2_ activation energies, where better ORR catalyst would attach O_2_ with binding energies close to−0.6 eV. We also calculated the O_2_ interaction on the Pt(111) surface, which is the industrial standard catalyst for the ORR. We find O_2_ binding energy of −0.65 eV and activation energy of 1.0 eV, which are close to those found for O_2_ on CoN5. To the best of our knowledge, this is the first time that Eb(O_2_) is calculated for CoN5 catalysts out of CoPc-Py-CNT and VB12 (Riquelme et al., 2018a). Eb(O_2_) is generally used as reactivity index in heterogeneous catalysis as well as in electrocatalysis. For example Norskov et al. reported volcano plot of activities (J, current density) of metal catalysts vs. Eb(OH) and suggested that in one side of the volcano we would find strong binding site vs. the opposite side of the volcano where weak binding site would occur or in other words 4-electron catalysts as Pt in one side vs. 2-electron catalyst as Au or Ag at the other side of the plot (i.e., the week binding side of the volcano; Viswanathan et al., 2012; Hansen et al., 2014). Zagal and Koper reported similar plots for MN4 complexes, distinguishing FeN4 and MnN4 as strong O_2_ binders while CoN4 and NiN4 would interact with O_2_ too weekly (Zagal and Koper, 2016). We recalculated the Eb(O_2_) for 8(2-Et-C_6_H_11_O)CoPc, CoPc, and 16(F)CoPc, and we found values that range between −0.32 eV to −0.38 eV (values resumed in Table 2). The performed calculations well agree with the previously estimated value of MN4 (Ruiz-Tagle and Orellana, 2010; Orellana, 2012, 2013). CoN5 would then bind O_2_ more similarly to Pt and Fe than to Au and Ag or CoN4 and NiN4 (i.e., more close to a 4-electron catalyst than to a 2-electron catalyst) and therefore their activity moves toward the apex in a theoretical volcano correlation (see Graphical Abstract).

**Table 1:** Resume of the total energy for 8(2-Et-C_6_H_11_O)CoPc-Py-CNT, CoPc-Py-CNT, and 16(F)-CoPc-Py-CNT with and without the O_2_ molecule adsorbed on the Co metal center for the allowed spin states.

***m* (μB)**	**8β(2-Et- C6H11O)CoP c-Py-CNT**	**O2-8β(2-Et-C6H11O)CoP c-Py-CNT**	**CoPc-Py-CNT**	**O2-CoPc-Py-CNT**	**16(F)-CoPc-Py-CNT**	**O2-16(F)CoPc-Py-CNT**
	*Etotal* (eV)	*Etotal* (eV)	*Etotal* (eV)	*Etotal* (eV)	*Etotal* (eV)	*Etotal* (eV)
0	E0 + 0.06	E0 + 0.04	E0 + 0.06	E0 + 0.08	E0 + 0.05	E0 + 0.04
1	E0	E0 + 0.08	E0	E0	E0	E0 +0.09
2	E0 + 0.03	E0	E0 + 0.04	E0 + 0.04	E0 + 0.04	E0
3	E0 + 0.10	E + 0.09	E0 + 0.15	E0 + 0.13	E0 + 0.15	E0 + 0.11

**Table 2:** Theoretical results for the magnetic moment (*m*), O_2_ binding energy (*E*_*bind*_), and O_2_ activation energy (*E*_*activ*_) for 8(2-Et-C_6_H_11_O)CoPc-Py-CNT, CoPc-Py-CNT, 16(F)-CoPc-Py-CNT, 8(2-Et-C_6_H_11_O), CoPc, and 16(F)CoPc in their lowest energy state.

**Compound**	***m* (μB)**	**O2 *Ebind* (eV)**	**O2 *Eactiv* (eV)**
8β(2-Et-C6H11O)CoPc-Py-CNT	1	−0.524	1.51
CoPc-Py-CNT	1	−0.593	1.35
16(F)CoPc-Py-CNT	1	−0.532	1.41
8β(2-Et-C6H11O)CoPc	1	−0.346	
CoPc	1	−0.382	
16(F)CoPc	1	−0.324	

### Electrochemical Characterization

**Figure 5:**
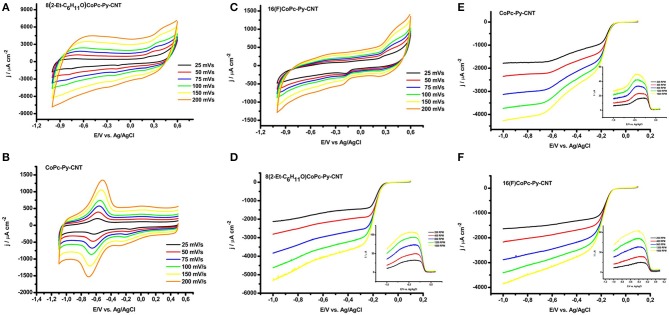
Cyclic voltammetry of **(A)** 8(2-Et-C_6_H_11_O)CoPc-Py-CNT, **(B)** CoPc-Py-CNT, **(C)** 16(F)CoPc-Py-CNT at 25, 50, 75, 100, 150, and 200 mV/s in N_2_ saturated NaOH 0.1 M. Linear sweep voltammetry of rotating ring disk electrodes at various rotating speeds (i.e., 200, 400, 800, 1,200, 1600 rpm) of **(D)** 8(2-Et-C_6_H_11_O)CoPc-Py-CNT, **(E)** CoPc-Py-CNT, **(F)** 16(F)CoPc-Py-CNT. Inset: oxidative current produce at the Pt ring electrode during the linear sweep voltammetry at the various rotation speeds. Conditions: potential scan rate of linear sweep experiments was 5 mV/s, Pt ring polarized at 0.6 V during chronoamperometry, O_2_ saturated NaOH 0.1 M.

Figures 5A–C illustrate a series of cyclic voltammograms measured at different scan rates (from 25 mv s^−1^ to 200 mV s^−1^) of graphite electrodes modified with CoN4-Py-CNT in N_2_ saturated 0.1 M NaOH solutions. The intensity of the anodic peaks is directly proportional to the scan rate velocity as expected for surface defined redox processes. Generally, CoN4 show an evident redox peak corresponding to the Co(II)/Co(I) transition at negative potentials (−0.715 V 8(2-Et-C_6_H_11_O)CoPc,−0.575 for CoPc,−0.4 V vs. Ag/AgCl for 16(F)CoPc), while the redox potential for the Co(III)/(II) redox peak is less evident (Barrera et al., 2006; Hebié et al., 2016; Venegasa et al., 2017). For 8(2-Et-C_6_H_11_O)CoPc-Py-CNT the redox peak for Co(II)/Co(I) is not well defined and appears at a potential of ≈−0.73 V (Figure 5A). For the CoPc-Py-CNT complex the Co(II)/Co(I) is very well defined at a potential of ≈ −0.58 V (Figure 5B). 16(F)CoPc-Py-CNT showed a not well defined redox peak at ≈ −0.5 V (Figure 5C). Apparently, for this complex, the Py back ligand acts as an electron-donating group. This might be an effect of the F groups located on the Pc ligand that essentially withdraw electron density from the axial Py group. These observations indicate that the combined electronic effects of the ligand and back ligand on the Co center are not additive, and are essentially push-pull electronic effects. Therefore, the effect of the axial coordination is opposite for the complexes with residues that are neutral or with a small electron-donating character and for the complex with a strong electron withdrawing residue as the perfluorinated compound. In Table 3 we display the Co(II)/(I) redox peak potentials for the various CoN5 as well as the surface concentration of the catalysts obtained from the integration of the Co(II)/(I) redox oxidation peaks from Figures 5A–C.

**Table 3:** Summary of the electrochemical results obtained for 8(2-Et-C_6_H_11_O)CoPc-Py-CNT (Blue), CoPc-Py-CNT (Black), and 16(F)CoPc-Py-CNT.

	**Onset (V)**	**N e-measuredat −0.5 V**	**Tafel Slope (V/decade)**	**E(Co(II)/(I) (V)**	**Coverage (mol/cm2)**	**TOF (S^**−1**^)**
8(2-Et-C6H11O)CoPc-Py-CNT	−0.07	3.3	−0.052	−0.73	2.94 × 10^−9^	0.4
CoPc-Py-CNT	0.07	3.2	−0.048	−0.58	1.52 × 10^−9^	0.4
16(F)CoPc-Py-CNT	−0.05	3.1	−0.048	−0.50	5.57 × 10^−10^	0.5

*Onset for the ORR; number of electrons transferred during the ORR; Tafel slope; Co(II)/(I) formal potential; concentration of the complex determined by the integration of the redox peaks of the Co(II)/(I) redox couples; turn over frequency (TOF) for the complex during ORR*.

In Figures 5D–F, we report the polarization curves for the ORR for electrodes modified with CoN5 in a 0.1 M NaOH solution saturated with O_2_ at various rotation speeds (from 200 to 1600 rpm). The ${\text{HO}}_{2}^{-}$ produced during the ORR at CoN5 modified graphite disks was monitored by polarizing the Pt ring electrode mounted in the same shuffle of the disk at 0.6 V (insets of Figures 5D–F). The onset for the ORR starts around −0.07 V for 8(2-Et-C_6_H_11_O)CoPc-Py-CNT and CoPc-Py-CNT while it starts around−0.05 V for 16(F)CoPc-Py-CNT. The production of ${\text{HO}}_{2}^{-}$ follows similar trends to the ORR (i.e., it decreases as the ORR catalytic current increases). At low overpotentials, in the mixed mass transport and kinetic controlled region, high amounts of ${\text{HO}}_{2}^{-}$ were produced with a maximum at−0.35 V for both 8(2-Et-C_6_H_11_O)CoPc-Py-CNT and 16(F)CoPc-Py-CNT. The CoPc-Py-CNT follows a slightly different process with a more pronounced ${\text{HO}}_{2}^{-}$ production with a peak at −0.3 and a dramatic decrease at −0.5 V. Anyway, the three catalysts present a bell shaped curve for ${\text{HO}}_{2}^{-}$ production, but for the CoPc-Py-CNT catalyst the difference between the activities of Co(I) and Co(II) toward the ORR is more pronounced probably because in the absence of residues at the periphery the differences between Co(I) and Co(II) are more accentuated.

**Figure 6:**
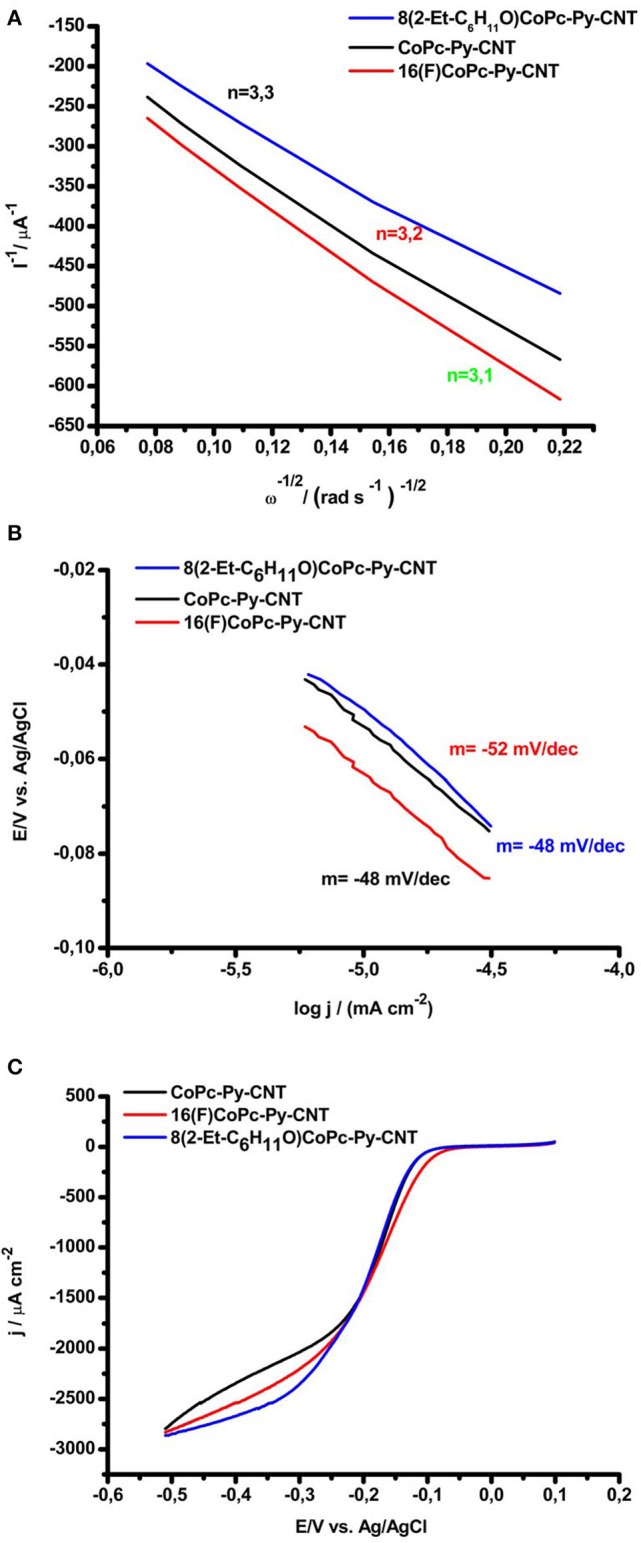
**(A)** Koutecky–Levich plots for O_2_ reduction for 8(2-Et-C_6_H_11_O)CoPc-Py-CNT (Blue), CoPc-Py-CNT (Black), and 16(F)CoPc-Py-CNT (red). **(B)** Tafel plots for O_2_ reduction for 8(2-Et-C_6_H_11_O)CoPc-Py-CNT (Blue), CoPc-Py-CNT (Black), and 16(F)CoPc-Py-CNT (red). **(C)** Linear sweep voltammetry comparison between 8(2-Et-C_6_H_11_O)CoPc-Py-CNT (Blue), CoPc-Py-CNT (Black), and 16(F)CoPc-Py-CNT (Red) during ORR at 800 rpm, scan rate 5 mv/s, O_2_ saturated NaOH 0.1 M.

In Figure 6A, the extrapolation of the experimental Koutecky–Levich (K–L) plots are presented. The values were extrapolated at −0.750 V. This potential was chosen for comparison reason, whereas the n of electrons would decrease at lower overpotentials (e.g., −0.3 V), where higher amounts of ${\text{HO}}_{2}^{-}$ are produced. The Koutecky–Levich regression shows that the reduction at CoN5 involves approximately 3.2 electrons (values for the 3 complexes are resumed in Table 3), which is in contrast to the typical 2-electron reduction process generally reported for Co attached directly to graphite and in the absence of an axial ligand. It should be noted that at higher overpotential the HO_2_ produced at the electrode surface decreases. This also suggests that the mechanism for the ORR is different from the CoN4. In Figure 6B the Tafel plots for the ORR for the CoN5 complexes are shown. The slopes determined from experiments presented values ranging from −0.048 V decade^−1^ to −0.052 V decade^−1^. For CoN4, slopes in the range of −0.060 V decade^−1^ were obtained (Zagal and Koper, 2016; Venegasa et al., 2017). Slopes close to −0.060 V as for CoN4 complexes suggest that the first step involves a fast electron transfer followed by a slow rate determining chemical step. In contrast, slopes of −0.040 V indicate that the first step involves a fast one-electron transfer followed by a slow one-electron transfer step. Generally slopes of −0.040 V are reported for Fe catalysts (Zagal and Koper, 2016; Venegasa et al., 2017). Slopes of ≈ −0.050 V decade^−1^ would, therefore, represent a situation in the middle where a mixed behavior is expected. Similar values were obtained before for VB12 adsorbed on CNT (Riquelme et al., 2018a). In Figure 6C we report, for comparison, the linear sweep voltammetry during ORR for the three complexes at the same rotation speed (i.e., 800 rpm) and at the same conditions. The onset for the ORR is very similar for the three complexes ranging from −0.07 V to −0.05 V. The mass transport limiting catalytic current is reached already at −0.3 V, showing the similarities between the three complexes when in the presence of the pyridine axial ligand.

In Table 3 we resume the turn over frequencies (TOF) estimated for CoN5 from the mass-related kinetic current density at −0.05 V. The comparison of the 3 TOF values reveals the similarity of the CoN5, which all can reduce O_2_ at very similar velocities. The fifth N coordination appears to play a strong role in the activities of CoN5. The effect of the axial coordination is stronger than the effect of the same plane residues that can be attached to the molecule. Therefore, the 3 complexes studied in this work, show similar activities toward the ORR and similar TOF values and energy binding values.

In a previous publication, we reported that the presence of the pyridine axial ligand as in CoPc-Py-CNT or as in VB12 increases the Co-O_2_ binding energy moving these complexes to the left-hand side of a volcano correlation, to the region of stronger M-O_2_ binding energy (Zagal and Koper, 2016; Riquelme et al., 2018a). New calculations performed on both CoN5 and CoN4 show that this is not completely true. If we consider the activities for CoN4 published in Zagal and Koper (2016); Venegasa et al. (2017) and we correlate them with the calculated O_2_ binding energy we can derive a linear correlation belonging to one side of a volcano correlation (Zagal and Koper, 2016). Also Zhou and collaborators showed an incremented activity toward the ORR increasing the electro-withdrawing force of the axial ligand coordinated to Co porphyrins (Zhou et al., 2019). This correlation would also be linear (i.e., only in one side the volcano). When in the presence of the back bond ligand Co phthalocyanines would bind O_2_ stronger and therefore the increment in the catalytic activities. CoN5 complexes would then move toward the apex of the volcano if compared to CoN4. Nevertheless, this would not be sufficient for correlating the complexes with the strong binding side of the volcano (see Graphical Abstract).

## Conclusions

We report for the first time the EPR and XPS spectra of the penta-coordinated complexes 8(2-Et-C_6_H_11_O)CoPc-Py-CNT, CoPc-Py-CNT, and 16(F)CoPc-Py-CNT. For 8(2-Et-C_6_H_11_O)CoPc-Py-CNT, EPR and XPS spectra show that the metal center is Co(II) high spin (S = 3/2) species, while for CoPc-Py-CNT it is Co(II) in a low spin configuration S = 1/2. For 16(F)CoPc-Py-CNT an intermediate spin (S = 1) Co(III) species are more probable. O_2_ binding energy were calculated and resulted to be very similar for the three complexes with values ranging from −0.52 to −0.59 eV. The CoN5 complexes were electrochemically characterized and their electrocatalytic activities toward the ORR were studied. Results show that the three complexes perform the ORR very similarly with a total of ≈ 3.2 electrons and Tafel slopes of ≈ −0.050 V decade^−1^. Therefore, CoN5 have a mixed behavior between a 2 and a 4-electron catalyst for the ORR. The pyridine back ligand act as an electron-withdrawing group for 8(2-Et-C_6_H_11_O)CoPc-Py-CNT and CoPc-Py-CNT, but as an electron-donating group for 16(F)CoPc because of the presence of the F residues located on the phthalocyanine ligand. Therefore, the combined electronic effects of the ligand and back ligand on the Co center are essentially push-pull electronic effects.

## Data Availability Statement

The datasets generated for this study are available on request to the corresponding author.

## Author Contributions

MV and JR performed electrochemical experiments. CA performed EPR experiments. JM performed XPS experiments. WO performed DFT calculation. JZ supervised the experimental and writing session. FT provided the main idea, writing, and supervision of the entire work.

### Conflict of Interest

The authors declare that the research was conducted in the absence of any commercial or financial relationships that could be construed as a potential conflict of interest.
